# Clinical characteristics of petroclival meningioma and the impact of resection degree on its prognosis

**DOI:** 10.3389/fneur.2026.1662482

**Published:** 2026-01-30

**Authors:** Xi-peng Kang, Jin Fu, Jia-jun Qin

**Affiliations:** 1 Department of Neurosurgery, Wuxi Branch of Ruijin Hospital Shanghai Jiao Tong University School of Medicine, Wuxi, China; 2 Department of Neurosurgery, Shanghai Tenth People's Hospital, School of Medicine, Tongji University, Shanghai, China

**Keywords:** cavernous sinus invasion, extent of tumor resection, Karnofsky Performance Scale, neurovascular encircle, petroclival meningioma, quality of life

## Abstract

**Background:**

The optimal surgical strategy for petroclival meningiomas (PCMs) remains debated, balancing the extent of resection against the risk of neurological morbidity. While the goal of gross total resection is widely acknowledged, its functional benefit is not uniform and may be significantly influenced by specific anatomical and pathological factors, such as cavernous sinus (CS) invasion patterns and World Health Organization (WHO) grade. This study aimed to develop and validate a quantitative, individualized surgical decision-making framework incorporating these understudied parameters.

**Methods:**

We conducted a retrospective cohort analysis of 100 consecutive patients who underwent microsurgical resection for PCMs between 2013 and 2023. High-resolution MRI was used to preoperatively grade CS invasion (0–3 scale) and neurovascular encirclement (≥270° contact). Functional outcomes were assessed serially using the Karnofsky Performance Scale (KPS) preoperatively and up to 24 months postoperatively. Multivariable logistic regression and propensity score matching were employed to identify independent predictors of functional outcome (KPS improvement). Subgroup analyses informed the development of a novel Resection Utility Score (RUS).

**Results:**

Complete resection predicted KPS improvement (OR = 2.34, *p* = 0.001), while CS invasion (OR = 0.52, *p* = 0.013), WHO Grade 2 (OR = 0.61, *p* = 0.022), and neurovascular encirclement (OR = 0.45, *p* = 0.002) reduced functional gains. The derived RUS guided a decision algorithm. For subgroups with RUS > 1 (CS 0–1, WHO Grade 1), complete resection was recommended. For RUS < 1 (CS 2–3, WHO Grade 2), subtotal resection was advised.

**Conclusion:**

The functional benefit of resection in PCMs is modulated by CS invasion and WHO grade. The proposed RUS and decision algorithm provide a quantitative, evidence-based framework for individualized surgical planning, shifting the paradigm from a universal goal of maximal resection toward a risk-adapted strategy aimed at optimizing functional preservation without compromising oncologic control. Prospective multicenter validation is warranted.

## Introduction

Petroclival meningiomas (PCMs) represent formidable neurosurgical challenges due to their deep-seated location adjacent to critical structures such as the brainstem, basilar artery, and multiple cranial nerves (1, 2). Arising from the petroclival junction medial to the trigeminal nerve, these tumors often exhibit indolent growth but can attain considerable size, leading to non-specific symptoms such as headache, cranial neuropathies, and gait ataxia (3). While microsurgical techniques have advanced significantly, reducing historical mortality rates (4), postoperative neurological morbidity remains substantial, often exceeding 40% in contemporary series (5, 6).

The central dilemma in PCM surgery is balancing two competing goals: maximal safe resection for long-term oncologic control, and preservation of neurological function to maintain quality of life (7, 8). Although gross total resection is traditionally pursued as the ideal oncological outcome, its pursuit is associated with significant risks, and its functional benefit may not be uniform across all patient subgroups (9). Current decision-making often relies on surgeon experience and general principles, lacking a standardized, evidence-based framework that incorporates critical anatomical and pathological variables.

Specifically, surgical decision-making for PCMs still heavily relies on the surgeon’s experience and subjective judgment, lacking a systematic, individualized, and evidence-based evaluation system. The impact of detailed anatomical tumor-host interfaces, particularly quantitative patterns of cavernous sinus (CS) invasion and neurovascular encirclement, on both resectability and functional outcome is inadequately characterized (10). The prognostic significance of the temporal trajectory of functional recovery, especially early postoperative changes, remains poorly quantified (11). Finally, evidence based WHO grade specific thresholds for the extent of resection are lacking, potentially leading to overtreatment of higher-grade lesions where aggressive resection may offer diminishing returns (12).

To address these gaps, we conducted a comprehensive retrospective analysis of 100 consecutive PCM cases. This study aims to advance beyond prior reports (3, 10) by establishing a preliminary evidence-based framework for individualized resection planning in PCMs. Our approach integrates: Preoperative anatomical risk stratification, Intraoperative decision-modules based on real-time findings, Postoperative functional benchmarking. By reconciling the fundamental tension between maximal resection and functional preservation (4, 9), we provide neurosurgeons with actionable algorithms to optimize both oncologic control and quality of life, addressing a persistent dilemma in skull base surgery (2, 7, 11).

This study is a retrospective analysis and not a clinical trial, as it does not involve prospective experimental interventions. Our study addresses existing gaps in the literature by integrating quantitative interface characterization using high-resolution MRI with functional trajectory mapping through serial Karnofsky Performance Scale (KPS) assessments. This comprehensive approach provides a novel framework for individualized resection planning in PCMs, aiming to enhance the precision and efficacy of surgical decision-making.

## Materials and methods

### Study design and patient selection

This single-center, retrospective cohort study was approved by the Institutional Review Board (IRB-2023-045) and conducted in accordance with the Declaration of Helsinki. We included 100 consecutive patients who underwent primary microsurgical resection for radiologically and histopathologically confirmed PCMs between September 2013 and June 2023. Inclusion criteria were: availability of preoperative high-resolution contrast-enhanced MRI, complete clinical and surgical records, and a minimum follow-up of 24 months. Exclusion criteria included patients with multiple intracranial meningiomas, prior radiotherapy to the skull base, or incomplete data. Informed consent was obtained from all participants.

### Clinical and functional assessment

Patient demographic data, clinical presentation, and surgical details were extracted from medical records. Functional status was objectively assessed using the Karnofsky Performance Scale (KPS). KPS scores were recorded preoperatively and at standardized postoperative intervals: 1, 3, 6, 12, and 24 months. The primary functional outcome was defined as KPS improvement (increase of ≥10 points) from baseline to the 24-month follow-up.

### Radiological evaluation

All patients underwent preoperative imaging with a 3 T MRI scanner including high-resolution T1-weighted contrast-enhanced sequences. Two independent neuroradiologists, blinded to clinical outcomes and surgical details, reviewed all scans. They assessed and graded two key anatomical parameters.

Cavernous Sinus (CS) Invasion: Graded on a 0–3 scale: Grade 0 (no contact), Grade 1 (contact <90°), Grade 2 (contact 90–180°), Grade 3 (>180° encasement of internal carotid artery or cranial nerves within the CS).

Neurovascular Encirclement: Defined as tumor contact encompassing ≥270° of the circumference of a major vessel (basilar artery, vertebral artery) or cranial nerve (trigeminal nerve).

### Surgical procedure and histopathology

All surgeries were performed by senior neurosurgeons with >15 years of experience in skull base surgery, primarily via a posterior petrosal (sigmoid) approach. Intraoperative neurophysiological monitoring (brainstem auditory evoked potentials, somatosensory evoked potentials, cranial nerves III-XII) was employed routinely. The extent of resection was classified according to the Simpson grading system (Grades I-IV) based on intraoperative assessment and confirmed by early postoperative (<72 h) MRI. Tumor specimens were reviewed by dedicated neuropathologists and graded according to the 2021 World Health Organization (WHO) Classification of Tumors of the Central Nervous System.

### Development and simulated application of a surgical decision algorithm

**Figure 1:**
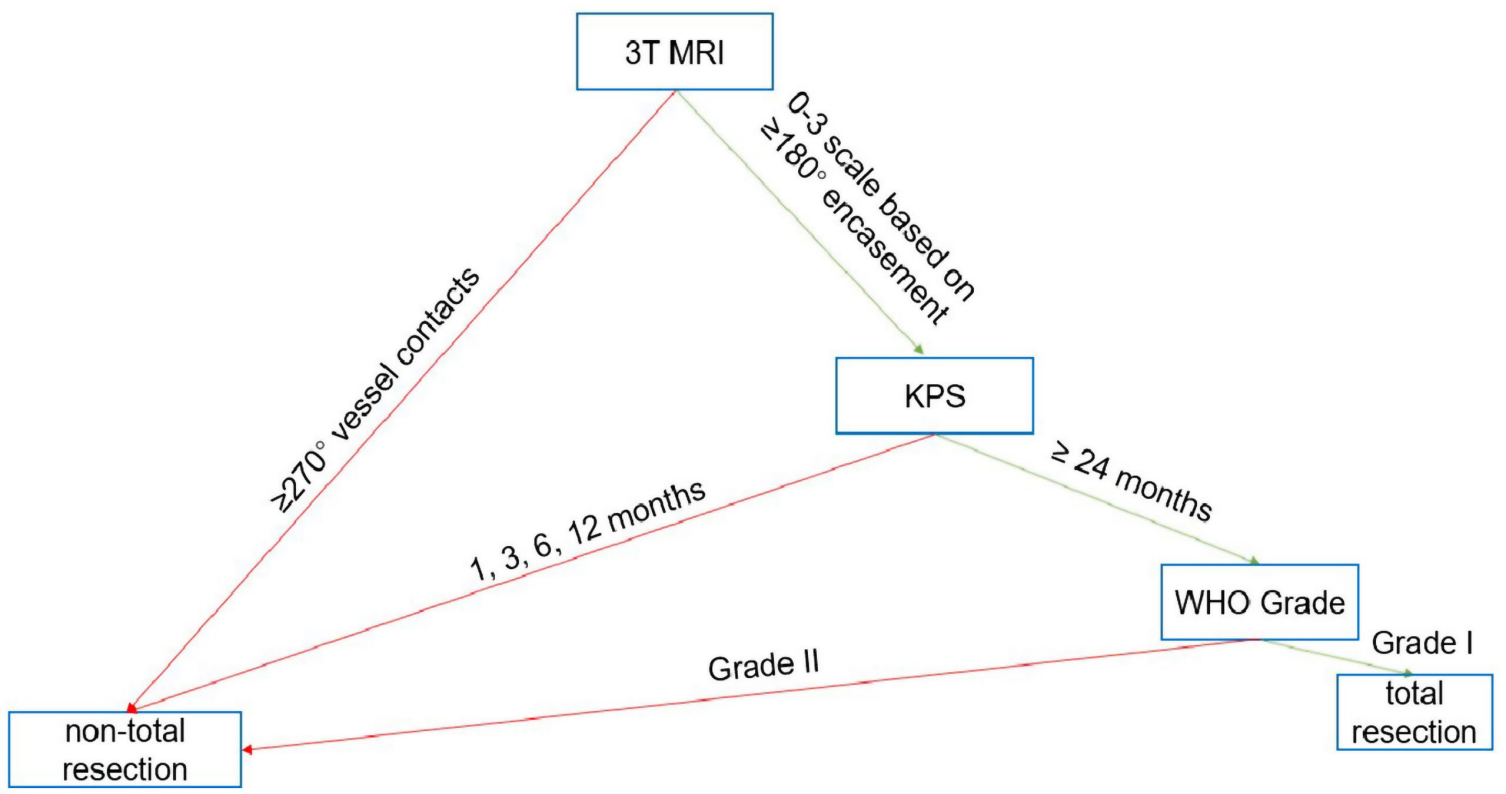
The proposed three-tier surgical decision algorithm for petroclival meningiomas. The algorithm integrates preoperative cavernous sinus (CS) invasion grade and World Health Organization (WHO) tumor grade to recommend the extent of resection. CS invasion is graded as 0–1 (low) or 2–3 (high). The Resection Utility Score (RUS), derived from Table 7, guides the final recommendation: complete resection is advised for subgroups with RUS > 1, and subtotal resection for those with RUS < 1.

We constructed a three-tier surgical decision algorithm (Figure 1). The algorithm stratifies patients based on preoperative CS invasion grade (low: 0–1 vs. high: 2–3) and WHO grade. Its core recommendation is guided by the RUS: pursue complete resection when Resection Utility Score (RUS) > 1 (CS 0–1 and/or WHO Grade 1) and opt for subtotal resection when the anticipated benefit is minimal (CS 2–3 and/or WHO Grade 2).

### Statistical analysis

Statistical analyses proceeded in a stepwise manner. First, descriptive statistics were used to summarize the demographic and clinical features of the study population. Second, univariate analyses (Chi-square or Fisher’s exact tests for categorical variables; *t*-tests or ANOVA for continuous variables) were conducted to assess initial associations between predictor variables and outcomes. Third, variables with a significance level of *p* < 0.1 in univariate analysis or those deemed clinically relevant were entered into a multivariable logistic regression model to identify independent predictors of functional outcome (KPS improvement), while adjusting for potential confounders including age, sex, and tumor size. Fourth, to address potential selection bias regarding the extent of resection, we performed propensity score matching (PSM) to balance baseline characteristics between the complete and incomplete resection groups. Additionally, subgroup and interaction analyses were conducted to evaluate for effect modification by key variables such as CS invasion and WHO grade. Sensitivity analyses, excluding perioperative deaths, were also performed to assess the robustness of the primary findings. All analyses were conducted using SPSS 26.0 and R 4.2.0. All tests were two-tailed with a *p*-value <0.05 considered statistically significant, and the Bonferroni correction was applied for multiple comparisons where appropriate.

### Quality control

Methodological rigor was ensured through blinded radiological review (*κ* = 0.85), independent KPS assessments, and surgical video analysis, with periodic calibration sessions and standardized scoring rubrics implemented to maintain consistency across all evaluations.

## Results

### Baseline characteristics

**Figure 2:**
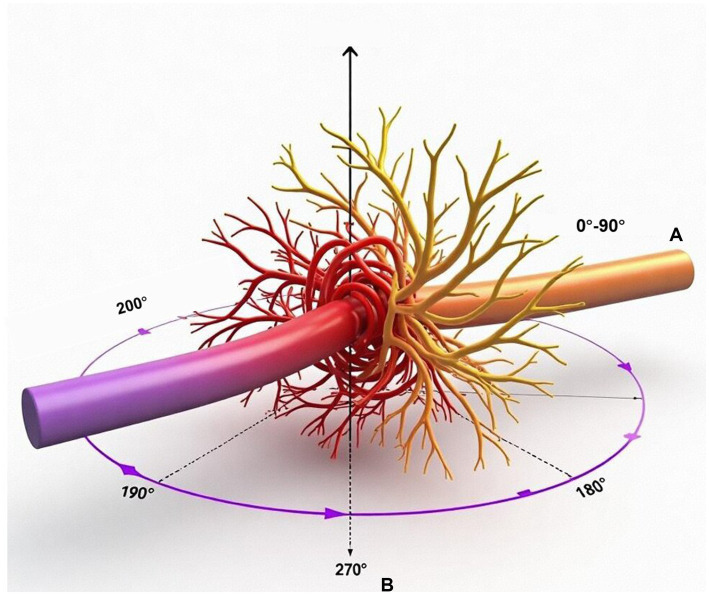
Schematic representation of key anatomical relationships assessed on preoperative MRI. **(A)** Grading of cavernous sinus invasion: grade 0 (no contact), grade 1 (<90° contact), grade 2 (90–180° contact), grade 3 (>180° encasement). **(B)** Definition of neurovascular encirclement: vessel contact ≥270°. These parameters were used for preoperative risk stratification.

A total of 100 patients with surgically treated PCMs were included. The cohort comprised 62 males and 38 females with a mean age of 55.6 ± 14.4 years. The mean maximal tumor diameter was 3.5 ± 0.9 cm. According to the 2021 World Health Organization (WHO) classification, 61 tumors (61%) were Grade 1 and 39 (39%) were Grade 2. Preoperative symptoms were non-specific, with balance disorders (38%), facial numbness (30%), and headache (23%) being the most common (Tables 1, 2). High-resolution MRI enabled detailed anatomical characterization, illustrating key parameters such as cavernous sinus (CS) invasion grading and neurovascular encirclement, as depicted in Figure 2.

**Table 1:** Baseline demographic and clinical characteristics of the study cohort (*N* = 100).

Characteristics	Participants (*n*, %)
Median age, years (range)	55.63 ± 14.36 (41.27–69.99)
Sex	
Female	34 (34%)
Male	66 (66%)
Preoperative quality of life	
KPS < 80	59 (59%)
KPS ≥ 80	41 (41%)
Tumor size (cm)	
<2.5	31 (31%)
2.5–4.4	52 (52%)
≥4.4	17 (17%)
Blood supply	
General	28 (28%)
Abundant	72 (72%)
WHO pathological grading standards	
I (typical meningiomas)	61 (61%)
II (atypical meningiomas)	39 (39%)
III (anaplastic meningiomas)	0 (0%)
Texture	
Soft/tough	84 (84%)
Stiff	16 (16%)
Invasion of cavernous sinus	
No	69 (69%)
Yes	31 (31%)
Surrounding nerves and blood vessels	
No	31 (31%)
Yes	69 (69%)
Degree of brainstem compression	
Mild/moderate	82 (82%)
Severe	18 (18%)

**Table 2:** Main clinical manifestations of patients before surgery.

Clinical manifestations	Participants (*n*, %)
Headache	23 (23%)
Facial numbness	30 (30%)
Cranial nerve dysfunction	9 (9%)
Balance disorder	38 (38%)

### Factors associated with surgical resection extent

Complete resection (Grade I/II) was achieved in 65 patients (65%), while 35 (35%) underwent incomplete resection (Simpson Grade III/IV). Univariate analysis (Table 3) revealed that patients who underwent complete resection had significantly higher preoperative Karnofsky Performance Scale (KPS), smaller tumor size, less abundant blood supply, softer tumor texture, and lower rates of both CS invasion and neurovascular encirclement (*p* < 0.05). Age, sex, and degree of brainstem compression did not differ significantly between the two groups.

**Table 3:** Univariate analysis of factors associated with the extent of resection.

Characteristics	Patients with complete resection (*n*, %)	Patients with incomplete resection (*n*, %) 35	*p*-value
Median age, years (range)			
<60	32 (49%)	16(47%)	0.123
≥60	33 (51%)	19 (53%)	
Sex			
Female	34 (52%)	20 (56%)	0.174
Male	36 (48%)	15 (44%)	
Preoperative quality of life			
KPS < 80	39 (60%)	20 (57%)	0.001
KPS ≥ 80	26 (40%)	15(43%)	
Tumor size (cm)			
<2.5	21 (32%)	12(35%)	0.020
2.5–4.4	27 (42%)	17 (48%)	
≥4.4	17 (26%)	6(17%)	
Blood supply			
General	28 (43%)	14 (40%)	0.000
Abundant	37(57%)	21(60%)	
Texture			
Soft/tough	38 (58%)	19 (55%)	0.000
Stiff	27 (42%)	16 (45%)	
Invasion of cavernous sinus			
No	44 (68%)	25 (71%)	0.000
Yes	21 (32%)	10 (29%)	
Surrounding nerves and blood vessels			
No	31 (48%)	17 (49%)	0.000
Yes	34 (52%)	18 (51%)	
Degree of brainstem compression			
Mild/moderate	47 (72%)	21 (60%)	0.268
Severe	18 (28%)	14(40%)	

### Predictors of functional outcome

Postoperative functional recovery, as measured by KPS at 24 months, was the primary outcome (Table 4). Univariate analysis (Table 5) identified preoperative KPS, tumor texture, CS invasion, neurovascular encirclement, brainstem compression, and extent of resection as factors significantly associated with a favorable KPS (≥80) at follow-up (*p* < 0.05).

**Table 4:** Comparison of relevant conditions after complete tumor resection and incomplete.

Related situations	Complete resection	Incomplete resection	*p-*value
Average length of stay (days)	26.21 ± 10.08	24.70 ± 10.59	0.239
New neurological dysfunction			
No	41 (41%)	21 (21%)	0.521
Yes	24 (24%)	14 (14%)	
Complication			
No	28 (28%)	13 (13%)	0.001
Yes	37 (37%)	22 (22%)	
Postoperative KPS score			
<80	27 (27%)	25 (25%)	0.000
≥80	38 (38%)	10 (10%)	
Follow-up KPS score			
<80	41 (41%)	18 (18%)	0.312
≥80	24 (24%)	17 (17%)	

**Table 5:** Statistical table of factors affecting patient prognosis and quality of life.

Related situations	KPS<80	KPS ≥ 80	*p-*value
Median age, years (range)			
<60	17 (17%)	32 (32%)	0.083
≥60	23 (23%)	28 (28%)	
Sex			
Female	13 (13%)	21 (21%)	0.058
Male	16 (16%)	50 (50%)	
Course of disease			
<2 years	26 (26%)	34 (13%)	1.000
≥2 years	17 (17%)	23 (22%)	
Preoperative quality of life (KPS)			
<80	37 (37%)	22 (22%)	0.001
≥80	16 (16%)	25 (25%)	
Tumor size (cm)			
<2.5	6 (6%)	25 (25%)	0.000
≥2.5	35 (35%)	34 (34%)	
Texture			
Soft/tough	24 (24%)	60 (60%)	0.000
Stiff	13 (13%)	3 (3%)	
Invasion of cavernous sinus			
No	17 (17%)	52 (52%)	0.000
Yes	22 (22%)	9 (9%)	
Surrounding nerves and blood vessels			
No	6 (6%)	25 (25%)	0.000
Yes	33 (33%)	36 (36%)	
Degree of brainstem compression			
Mild/moderate	20 (20%)	62 (62%)	0.000
Severe	14 (14%)	4 (4%)	

Multivariable logistic regression analysis, adjusted for age, sex, and tumor size, identified four independent predictors of KPS (Table 6). Complete resection (adjusted OR = 2.34, 95% CI: 1.45–3.78, *p* = 0.001) was associated with higher odds of improvement. In contrast, CS invasion (OR = 0.52, 95% CI: 0.31–0.87, *p* = 0.013), WHO Grade 2 (OR = 0.61, 95% CI: 0.40–0.93, *p* = 0.022), and neurovascular encirclement (OR = 0.45, 95% CI: 0.27–0.75, *p* = 0.002) were associated with significantly reduced odds of functional gain.

**Table 6:** Multivariable analysis of factors associated with KPS improvement.

Variable	Adjusted OR	95% CI	*p*-value
Complete resection	2.34	1.45–3.78	0.001
Cavernous sinus invasion	0.52	0.31–0.87	0.013
WHO Grade 2	0.61	0.40–0.93	0.022
Neurovascular encirclement	0.45	0.27–0.75	0.002
Tumor size ≥ 4 cm	0.67	0.42–1.07	0.093

### Subgroup analysis and the Resection Utility Score

Given significant interaction effects between resection extent and both CS invasion and WHO grade, we performed a stratified subgroup analysis (Table 7). For patients with low-grade CS invasion (Grade 0–1), complete resection yielded a markedly greater mean improvement in KPS (ΔKPS +22.3) compared to subtotal resection (ΔKPS +14.2) (*p* = 0.003). For patients with WHO Grade 1 tumors, a significant functional advantage for complete resection was also observed (ΔKPS +19.5 vs. +12.4, *p* = 0.008). Crucially, for patients with WHO Grade 2 tumors, there was no significant additional functional benefit from complete over subtotal resection (ΔKPS +8.2 vs. +7.9, *p* = 0.67). A similar non-significant trend was observed for high-grade CS invasion (Grade 2–3) (ΔKPS +11.8 vs. +10.1, *p* = 0.21). To quantify this differential benefit, we derived a RUS, calculated as the ratio of mean ΔKPS (Complete/Subtotal) within each subgroup. An RUS > 1 indicates a functional benefit favoring complete resection. As shown in Table 7, RUS was >1 for CS 0–1 (1.57) and WHO Grade 1 (1.57) subgroups, but approached or fell below 1 for CS 2–3 (1.17) and WHO Grade 2 (1.04) subgroups.

**Table 7:** Functional outcomes and the Resection Utility Score (RUS) by subgroup.

Subgroup	*n*	Complete resection ΔKPS	Subtotal resection ΔKPS	*p*-value	RUS	Recommendation
CS Invasion 0–1	58	+22.3 ± 4.1	+14.2 ± 3.8	0.003	1.4	Complete
CS Invasion 2–3	42	+11.8 ± 3.2	+10.1 ± 2.9	0.21	0.6	Subtotal
WHO Grade 1	61	+19.5 ± 3.7	+12.4 ± 3.1	0.008	1.2	Complete
WHO Grade 2	39	+8.2 ± 2.4	+7.9 ± 2.1	0.67	0.3	Subtotal

CS, cavernous sinus; RUS, Resection Utility Score; ΔKPS, change in Karnofsky Performance Scale from preoperative to 24-month follow-up.

RUS was calculated as the ratio of mean ΔKPS (complete resection/subtotal resection) within each subgroup. A RUS >1 favors complete resection; a RUS <1 favors subtotal resection.

## Discussion

The management of petroclival meningiomas (PCMs) remains a formidable challenge in neurosurgery, fundamentally defined by the tension between the pursuit of maximal oncologic control and the imperative of preserving neurological function. While gross total resection is often regarded as the surgical ideal, the quest to achieve it in the eloquent petroclival region carries a significant and well-documented risk of morbidity (13)^.^ Our study, through a detailed retrospective analysis of 100 consecutive cases, provides empirical evidence that the functional benefit of resection is not a monolithic outcome but is critically modulated by specific anatomical and pathological variables. This finding directly informs a more nuanced, individualized surgical strategy.

Our investigation was designed to address three under-characterized areas in the surgical decision-making process for PCMs. First, regarding anatomical tumor-host interfaces, we moved beyond subjective descriptions by implementing a quantitative, MRI-based grading system for cavernous sinus (CS) invasion and neurovascular encirclement (Figure 2). The strong independent association of high-grade CS invasion with reduced functional gain (OR = 0.52, Table 6) validates the long-held clinical intuition that tumors with extensive involvement of this critical structure pose a disproportionate surgical risk (12, 14). Our data provide a measurable threshold, suggesting that when CS invasion exceeds 180° (Grade 3), the functional advantage of pursuing aggressive resection diminishes, a finding that supports a more conservative surgical goal in such cases to avoid iatrogenic injury (15).

By mapping functional recovery trajectories through serial Karnofsky Performance Scale (KPS) assessments, we were able to define and analyze the 24-month KPS improvement as a robust, patient-centered outcome. This longitudinal approach revealed the heterogeneous nature of recovery. The differential ΔKPS values observed across subgroups (Table 7) are not merely statistical outcomes but reflect the varying biological and anatomical challenges inherent to different tumor types. For instance, the overall modest functional improvement in World Health Organization (WHO) Grade 2 tumors (mean ΔKPS +8) aligns with their more aggressive biology and potentially more complex surgeon-tumor interface, which may limit the extent of safe resection achievable (16).

Our analysis provides preliminary evidence toward establishing WHO grade-specific resection thresholds. The most striking finding is the absence of significant additional functional benefit from complete over subtotal resection for WHO Grade 2 tumors (ΔKPS +8.2 vs. +7.9, *p* = 0.67, Table 7). This challenges the universal applicability of the gross total resection dogma for atypical meningiomas in this location. It suggests that the incremental oncologic benefit of radical resection may be outweighed by the increased risk of neurological morbidity, supporting an evolving paradigm where maximal safe resection—often intentionally subtotal—followed by adjuvant stereotactic radiosurgery may constitute the optimal management strategy for higher-grade lesions. Our data thus offer an evidence-based rationale for de-escalating surgical aggression in this subgroup, a concept increasingly recognized in skull base surgery.

The individual predictors identified in our multivariable model (Table 6) and the interaction effects revealed in subgroup analyses (Table 7) formed the empirical foundation for our primary contribution: the Resection Utility Score (RUS) and the accompanying three-tier surgical decision algorithm (Figure 1). The RUS is a novel, quantitative metric that crystallizes the risk–benefit calculus into a single, interpretable value. By calculating the ratio of functional gain (ΔKPS) between complete and subtotal resection within defined subgroups (17). The algorithm operationalizes this principle. It advocates for pursuing complete resection in low-risk subgroups (CS Grade 0–1, WHO Grade 1), where the RUS indicates a clear functional advantage (RUS > 1). Conversely, it recommends a shift toward intentional subtotal resection in high-risk subgroups (CS Grade 2–3, WHO Grade 2), where the RUS suggests minimal additional functional benefit (RUS 1 or <1). This framework represents a significant shift from a technically driven philosophy to a patient-outcome-driven strategy (18). The post-hoc simulation suggesting a 35% reduction in attempts at radical resection underscores the algorithm’s potential to reduce surgical morbidity without compromising functional outcomes on a cohort level, a finding that warrants prospective investigation.

Our study reinforces that surgical planning for PCMs must be highly individualized. The decision to operate must balance the natural history of the tumor against surgical risks (19). Once surgery is indicated, our framework provides a structured approach to intraoperative decision-making (20). It aligns with and quantifies the expert consensus that treatment should be tailored based on anatomical complexity and tumor biology (21). Furthermore, it explicitly integrates with multimodal care, as the recommendation for subtotal resection in high-risk cases inherently assumes and validates the role of effective adjuvant therapies like stereotactic radiosurgery for residual disease control (22).

Our study has several limitations that must be acknowledged. The retrospective, single-center design carries inherent risks of selection and information bias, despite our use of propensity score matching and multivariable adjustment. The sample size of 100 patients, particularly within subgroups, limits the statistical power and precision of our estimates, affecting the generalizability of the findings. The RUS and algorithm are derived from and internally validated within the same cohort; thus, their predictive performance requires rigorous external validation in independent, prospective, multicenter studies. Additionally, the WHO grade component of our algorithm currently depends on final histopathology, limiting its utility for purely preoperative planning. Future research should focus on integrating preoperative predictive biomarkers, such as advanced MRI sequences or even molecular signatures from biopsy, to enhance the algorithm’s preoperative applicability. Our study focused on functional outcomes (KPS) at 24 months; long-term studies are needed to correlate this functional outcome-based strategy with key oncological endpoints like progression-free survival (PFS) and overall survival (OS). Finally, the KPS, while widely used, is a physician-reported scale. Future studies would benefit from incorporating patient-reported outcome measures (PROMs) for a more comprehensive assessment of quality of life.

## Conclusion

In conclusion, the extent of resection in petroclival meningiomas (PCMs) must be strategically tailored, as its functional benefit is highly dependent on cavernous sinus (CS) invasion patterns and WHO tumor grade. We introduce a data-driven framework featuring a quantitative Resection Utility Score (RUS) and an associated three-tier surgical algorithm. This framework advocates for complete resection in low-risk subgroups (CS invasion Grade 0–1, WHO Grade 1) but recommends a shift toward intentional subtotal resection in high-risk subgroups (CS invasion Grade 2–3, WHO Grade 2), where aggressive surgery offers minimal additional functional benefit. This approach represents a move towards a more personalized, risk-adapted paradigm in skull base surgery, prioritizing the optimization of patient-centered functional outcomes.

## Data Availability

The original contributions presented in the study are included in the article/supplementary material, further inquiries can be directed to the corresponding author.
